# CTO: a Community-Based Clinical Trial Ontology and its Applications in PubChemRDF and SCAIView

**Published:** 2020-09

**Authors:** Asiyah Yu LIN, Stephan GEBEL, Qingliang Leon LI, Sumit MADAN, Johannes DARMS, Evan BOLTON, Barry SMITH, Martin HOFMANN-APITIUS, Yongqun Oliver HE, Alpha Tom KODAMULLIL

**Affiliations:** a Center for Devices and Radiological Health, FDA, Silver Spring, MD, USA; b Department of Bioinformatics, Fraunhofer Institute for Algorithms and Scientific Computing (SCAI), Schloss Birlinghoven, Sankt Augustin, Germany; c National Center for Biotechnology Information, National Library of Medicine, National Institutes of Health, Bethesda, MD 20894, USA; d University of Michigan Medical School, Ann Arbor, Michigan, USA; e National Center for Ontological Research, Buffalo, NY, USA; f University at Buffa1o, Buffalo, NY, USA

**Keywords:** clinical trial, clinical trial registry, ontology, BFO, COVID-19

## Abstract

Driven by the use cases of PubChemRDF and SCAIView, we have developed a first community-based clinical trial ontology (CTO) by following the OBO Foundry principles. CTO uses the Basic Formal Ontology (BFO) as the top level ontology and reuses many terms from existing ontologies. CTO has also defined many clinical trial-specific terms. The general CTO design pattern is based on the PICO framework together with two applications. First, the PubChemRDF use case demonstrates how a drug Gleevec is linked to multiple clinical trials investigating Gleevec’s related chemical compounds. Second, the SCAIView text mining engine shows how the use of CTO terms in its search algorithm can identify publications referring to COVID-19-related clinical trials. Future opportunities and challenges are discussed.

## 1. Introduction

Clinical trials are research studies conducted on human participants to evaluate medical, surgical, or behavioral interventions involving investigational drugs, devices, diagnostic products, treatments and the like. Well-designed and well-conducted clinical trials are considered the best source of evidence to evaluate the efficacy and safety of medical interventions^[1]^. As human subjects are involved, clinical trials are subject to the Declaration of Helsinki requirement to the effect that any interventional clinical trial should be reported in a timely fashion^[2]^. In 2015, WHO reaffirmed the ethical imperative of reporting results of clinical trials, and outlined the steps for linking clinical trial registries to their published results^[3]^.In the USA, Title VIII of the Food and Drug Administration (FDA) Amendments Act of 2007 (FDAAA) mandates sponsors and others responsible for certain clinical trials of FDA-regulated drug, biologic, and device products to register their studies and report summary results in ClinicalTrials.gov, which is managed by the National Library of Medicine (NLM)^[4]^. Similarly, the European Union Clinical Trials Directive 2001/20/EC and European clinical trials regulation (No 536/2014) requires research transparency and openness to the public^[5]^.

Numerous clinical trial registries have been established by governments, non-governmental organizations, universities, as well as commercial and nonprofit entities. Examples include WHO’s International Clinical Trials Registry Platform (ICTRP, https://www.who.int/ictrp/en/), ClinicalTrials.gov in the US, the European Union Clinical Trials Register (https://www.clinicaltrialsregister.eu/), and other nation- or country-specific centralized clinical trial registries. Different clinical trial registries apply different clinical trial standards and use different vocabularies, and this results in siloed clinical trial reporting and heterogeneity of reported data. The ICTRP comprehends 20 district registry platforms including ClinicalTrials.gov. To facilitate data transformation, the WHO and the International Committee of Medical Journal Editors (ICMJE) developed the WHO/ICMJE ClinicalTrials.gov Cross Reference^[6]^, which provides mappings between WHO’s 24 data elements and the Registration Data Elements used in ClinicalTrials.gov. Yet, even in this mapping document we find the ambiguous use of similar terms and mismatched granularities of terminologies.

Ontologies have been one of the solutions proposed as integrative framework for the harmonization of terminologies in cases such as this. An ontology is a representation of the types of entities in a given domain and of the relations between them and including also a set of definitions of its terms and relational expressions. The ontology provides a controlled vocabulary, and computer-interpretable definitions of its terms using the OWL description logic which at the same time establishes a formal semantics. The ontology provides also human-readable natural language definitions to guide both developers and users of the ontology. An ontology-based data integration framework thereby provides an intermediate translational layer to harmonize different terminologies without creating new common data elements. It also provides an extendable and sustainable solution to maintain machine-readable mappings among multiple terminologies. To achieve these goals it is important to use a community-based strategy to develop the ontology in order to promote maximum community involvement in development of the ontology and in usage of the ontology to create annotations^[7]^.

The Open Biological and Biomedical Ontologies (OBO) Foundry is a community of ontology developers committed to the collaborative and coordinated realization of these goals, and they have established a set of principles for ontology development, including use of the realism-based top-level Basic Formal Ontology (BFO) (http://basic-formal-ontology.org/), which has been adopted as top level by over 200 ontology development groups in the biomedical domain^[8]^, and which has recently been approved as an ISO/IEC 21838-2 standard (https://www.iso.org/standard/71954.html)

The principles of the OBO Foundry are designed to foster the compatibility and interoperability of its member biomedical ontologies, and therefore we use OBO ontologies as the base for the development of the Clinical Trial Ontology (CTO) with the goal of formally representing and integrating all terminologies used in clinical trials.

## 2. Methodology

### 2.1. Development of Clinical Trial Ontology (CTO)

More specifically, the goal of CTO is to align and expand all terminologies used by clinical trial registries in order to represent clinical trial data at multiple levels of granularity. As a prototype, we started by ontologizing the small set of 24 terms outlined in the WHO/ICMJE – ClinicalTrials.gov Cross Reference^[6]^. Both top-down and bottom-up approaches were applied for this purpose. For our top-down approach we used BFO as the top level of CTO itself, which then largely reuses BFO-based OBO ontologies such as the Ontology for Biomedical Investigation (OBI), the Information Artifact Ontology (IAO), and the Ontology for Precision Medicine and Investigation (OPMI)^[9]^. We reuse also the previously developed Clinical Trial Ontology-Neurodegenerative Diseases (CTO-NDD)^[10]^, which was re-engineered to be BFO compliant. Domain experts, ontologists, bioinformaticians, and software developers met weekly to discuss the term definitions and hierarchy. The team investigated and vetted the terms and definitions from all existing clinical trial-related terminologies, including but not limited to: CDISC, NCIt, SNOMED CT, and OCRe. Besides the definitions provided by WHO and ClinicalTrials.gov, we employed terminologies such as the NCI Thesaurus where needed to complement the textual definitions of terms in CTO. The PICO framework^[11,12]^ was adopted to model the key terms used in the clinical trial domain.

The Protégé OWL editor was used to construct the ontology, and the OntoFox tool^[13]^ was used to import and reuse terms from other OBO Foundry ontologies. For the Gleevec PubChemRDF use case, the Cmap tool (https://cmap.ihmc.us/) was used to draw pattern designs, and the RDFLib tool was used to create RDF graphs. For the SCAIView text mining use case, the tagger for the clinical trial identifiers and registry terms was implemented using the Apache Unstructured Information Management Architecture (UIMA) (https://uima.apache.org) and Apache UIMA Ruta™ (https://uima.apache.org/ruta.html). A COVID-19 related literature corpus was processed in a parallel mode with Apache Spark (https://spark.apache.org/) running on a single node of four Intel® Xeon® Platinum 8160 CPUs with 96 cores (192 threads) and 1.5TB RAM in the inhouse HPC cluster.

### 2.2. Ontology Deposition and License

As an open source community-based development project, CTO is available at GitHub (https://github.com/ClinicalTrialOntology/CTO) with Creative Commons Attribution 4.0 International Public License (CC-BY) license. CTO is listed as a reference ontology in OBO foundry, and can be searched via the Ontobee interface: http://www.ontobee.org/ontology/CTO. In addition, CTO is also available via the BioPortal repository.

## 3. Results

### 3.1. CTO ontology architecture

**Figure 1.**
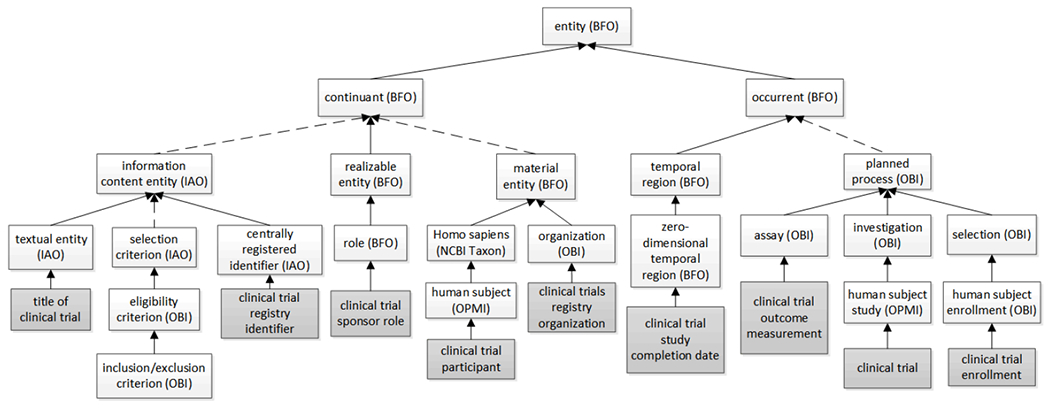
The upper hierarchical architecture of CTO aligned to BFO. CTO specific terms are shaded. Solid arrows indicate direct child-mother relations, dotted arrows indicate hierarchies of indirect child-mother relations.

CTO is a hybrid of multiple OBO Foundry ontology terms and clinical trial-specific terms, descending from BFO through a process of specialization (passing from the more general to the less general by means of two-part definitions, as outlined in^[14]^), and with OBO ontology terms in the middle and CTO specific terms at the bottom (Figure 1).

First, CTO terms relating to processes are included under ‘occurrent’ (BFO). For example, some processual terms are listed under ‘planned process’ (OBI); this includes the ‘clinical trial’ itself which is included as a child of ‘human subject study’ (OPMI), which is itself a child of ‘investigation’ (OBI); and the ‘clinical trial enrollment’ is a subclass of ‘human subject enrollment’ (OBI), which is a child term of ‘selection’ (OBI). Another set of processual CTO terms are date-related, as in: ‘clinical trial study completion date’, which is a child of ‘zero-dimensional temporal region’ (BFO), which is itself a child of ‘temporal region’ (BFO).

Second, examples of CTO terms representing what BFO calls continuants are:

1. terms under BFO ‘material entity’ such as: ‘clinical trials registry organization’ – a subclass of ‘organization’ (OBI), and ‘clinical trial participant’ – a subclass of ‘human subject’ (OPMI);
2. terms under BFO ‘realizable entity’ such as ‘clinical trial sponsor role’, a subclass of ‘role’ (BFO); 3) terms under IAO ‘information content entity’ such as ‘clinical trial registry identifier’, the latter a subclass of ‘centrally registered identifier’ (IAO), and also: ‘title of clinical trial’ – a subclass of ‘textual entity’ (IAO). Terms such as ‘inclusion criterion’ and ‘exclusion criterion’ have been defined in OBI and were directly imported into CTO.

### 3.2. CTO modeling of the PICO elements for clinical trials

**Figure 2.**
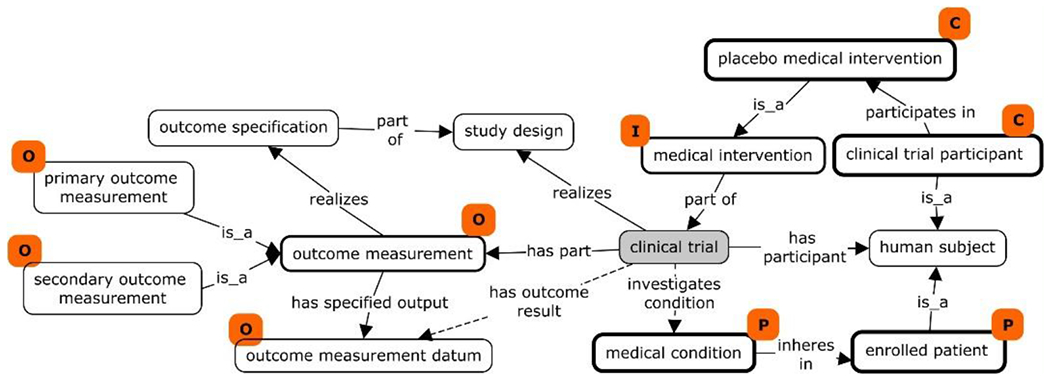
CTO representation of PICO elements in a scenario of patients treated with a medical intervention compared to healthy enrollees treated with placebo. PICO elements corresponding to ontological classes are marked with orange tags, and the principal classes representing PICO elements are in boxes marked by stronger borders. Shortcut relations are represented using dotted lines.

PICO is a knowledge representation framework designed to allow the formulation of the clinical research questions that arise in evidence based medicine^[11,12]^. Here ‘P’ stands for “Population of interest/Patient/Problem”, ‘I’ for “Intervention exposure to be considered–treatments/tests”, ‘C’ for “Control/comparison intervention treatment/placebo/standard of care” and ‘O’ for “Outcome of interest”. We started with the representation of a clinical trial by modeling these PICO elements (Figure 2). With ‘human subject’ as its participates, a ‘clinical trial’ investigates a ‘medical condition’ (P), which inheres in an ‘enrolled patient’ (P). The shortcut relation ‘*investigates condition*’ was created to establish a direct link between a clinical trial and a condition or disease. The ‘medical intervention’ (I) is a planned process that forms a part of a ‘clinical trial’. The comparison group or treatment in a clinical trial is represented by the term ‘placebo medical intervention’ (C), which is itself a child of ‘medical intervention’. A ‘clinical trial participant’ who participates in the ‘placebo medical intervention’ is a member of a comparison group (C). The ‘clinical trial’ has part ‘outcome measurement’ (O), which has subclasses: ‘primary outcome measurement’ (O) and ‘secondary outcome measurement’ (O). As a planned process, the specific output of the process of ‘outcome measurement’ is an ‘outcome measurement datum’ (O). The ‘outcome measurement’ realizes and concretizes the ‘outcome specification’, which is a part of the ‘study design’. A ‘clinical trial’ realizes the plan specified in its ‘study design’, which is itself created during the clinical trial planning phase. To facilitate data integration, another shortcut relation ‘*has outcome result*’ was created to link ‘clinical trial’ to ‘outcome measurement datum’.

CTO defines the ‘study design’ as a specification created during the planning phase of a clinical trial. The ‘clinical trial’ realizes the successive parts of the plan specified in the ‘study design’. For example, a ‘Random controlled double blinded clinical trial’ realizes ‘randomized allocation’, ‘double blinded masking design’ and ‘case-control comparison design’.

### 3.3. CTO’s treatment of required data elements linking ClinicalTrials.gov and WHO

As we mentioned above, the WHO/ICMJE – ClinicalTrials.gov Cross Reference document contains a list of 24 terms from the WHO Trial Registration Data Set (Version 1.3.1) mapped to some 50 terms whose usage is required by ClinicalTrials.gov. 12 (50%) of these WHO terms, mapped to 15 CTO terms, pertain to the identifiers, titles, dates, sponsors, responsible contacts, and country jurisdictions of the clinical trial registration. These terms are thus important data elements for clinical trial registry data governance.

#### 3.3.1. CTO’s treatment of clinical registry has trials registry identifiers

As a database, each registry assigns its own identifiers to the clinical trial records stored in the registry. In CTO, the ‘clinical trial registry identifier’ is a class instantiated by actual IDs (instances) of each clinical trial record in a given registry. Some registries are a combination of several registries, therefore, multiple identifiers become subclasses of one identifier ; this applies, for example, to the ‘Japan clinical trial identifier’, which has ‘jRCT clinical trial identifier’, ‘JMACCT clinical trial identifier’ and ‘UMIN-CTR clinical trial identifier’ as its subclasses. Another situation arises with the use of both ‘primary’ and ‘secondary’ registry identifiers. For example, WHO’s ICTRP accepts clinical trial records that are submitted from other registries and assigns each record an ICTRP unique identifier. In this case, ICTRP considers its own identifier as the ‘primary registry identifier’, and other IDs associated with the submitted record as ‘secondary registry identifiers’. To deal with such cases CTO defines ‘primary’ and ‘secondary’ identifier roles. CTO contains all known registry identifiers that are considered as ‘primary’ in WHO ICTRP.

#### 3.3.2. CTO’s treatment for dates

The CTO term ‘clinical trial start date’ is a child of ‘study start date’ (OPMI). Textually, the ClinicalTrials.gov ‘Study Start Date’ data element is the same as the OPMI ‘study start date’. However, they are different semantically. This is because ‘study’ has a wider scope than ‘clinical trial’, since the former may be an animal study, where the latter is limited to studies involving only humans. Hence, in CTO, the ‘clinical trial start date’ has exact synonyms ‘Study Start Date’ from ClinicalTrials.gov and the ‘date of first enrollment’ from WHO’s ICTRP. Other date classes in CTO include: ‘clinical trial primary completion date’ and ‘clinical trial study completion date’. All such terms are asserted as children of ‘zero-dimensional temporal region’ (BFO). A relation ‘occurs on’ obtains between the processes represented by the process terms in CTO and corresponding temporal regions^[15]^.

#### 3.3.3. CTO’s treatment for stakeholders’ roles related to a clinical trial

Normally, a clinical trial requires multi-stakeholder engagement over a long period of time. Sponsors, both primary and secondary, are either financially responsible for supporting the trial, or responsible for initiating or managing it. Investigators and collaborators are scientists or clinicians who carry out the clinical study at clinical site(s). Multiple roles were created in the CTO hierarchy under the OBI term: ‘investigation agent role’: the ‘investigation collaborator role’, the ‘investigator role’ with its children, and the ‘responsible party role’, ‘clinical trial sponsor role’ and its children terms. The contact information for a clinical trial is required in the registry. Many different contact terms were created in CTO, including ‘central contact person’, ‘facility contact’, ‘contact for public queries’, and ‘contact for scientific queries’. The ‘contact person role’ was created in CTO as parent to handle these terms. The ‘contact person information’ is asserted in CTO as a subclass of ‘information content entity’ (IAO), and the ‘is about’ relation used to link it to ‘contact person’.

### 3.4. Gleevec PubChemRDF Use Case: Linking PubChemRDF to ClinicalTrials.gov

PubChem is an open chemical information resource at the U.S. National Center for Biotechnology Information (NCBI)^[16]^. PubChemRDF is a semantic version of the content of the PubChem corpus (https://pubchemdocs.ncbi.nlm.nih.gov/rdf), and it consists of over 80 billion triples organized into more than 15 subgraphs including Compound, Protein, BioAssay, Pathway and Reference. Besides the interlinks of subgraphs, PubChemRDF can also link to external semantic resources, such as UniProt RDF, MeSH RDF, and Wikidata. Although PubChem has parsed and linked the molecular entities in clinical trial data to individual chemical compounds including drugs, the PubChemRDF does not currently link to clinical trial data due to the lack of an RDF graph that would serve this purpose.

**Figure 3.**
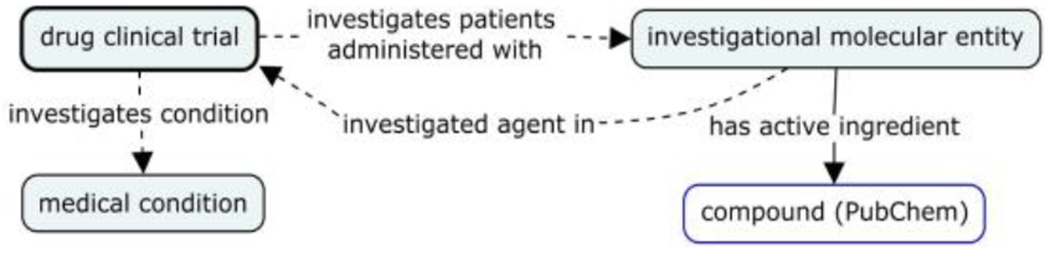
A simplified representation of drug clinical trial data linking to key data element in ClinicalTrials.gov and PubChem compound data

In order to take full advantage of the existing PubChemRDF ecosystem, however, it would be very useful to be able to create direct links between clinical trials, their disease targets, and the associated investigational drugs as referenced in PubChemRDF. Figure 3 shows a simplified diagram using multiple shortcut relations to allow the trade-off between a formal ontology representation of CTO and the RDF graph created therefrom. A ‘drug clinical trial’ investigates patients who are administered with an ‘investigational molecular entity’, which ‘*has active ingredient*’ that is a ‘compound’ in PubChem. The reverse relation of ‘*investigates patients administered with*’ is ‘*investigated agent in*’, which provides a direct link from an ‘investigational molecular entity’ to a clinical trial. The ‘investigational molecular entity’ corresponds to the ‘Drug’ and ‘Biologics’ data elements from the intervention type list defined in ClinicalTrials.gov’s XML schema (https://clinicaltrials.gov/ct2/html/images/info/public.xsd). The shortcut relation from ‘drug clinical trial’ to the ‘medical condition’ born by the enrolled patient is ‘*investigates condition*’ (shown in Figure 3). The shortcut relations can be implemented using OWL’s property chain, thereby providing an efficient solution for PubChemRDF without sacrificing the semantics built in the ontology.

**Figure 4.**
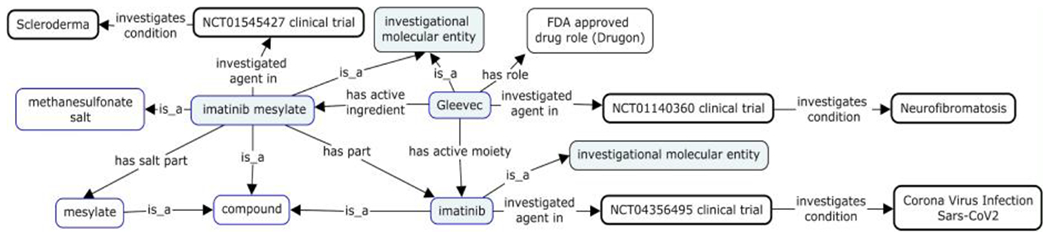
Three clinical trials investigating Gleevec, imatinib mesylate, and imatinib for three different medical conditions. Boxes with blue lines are entities in PubChem scope. Boxes with black heavy lines are entities extracted from ClinicalTrials.gov. Light blue colored boxes denote ‘investigational molecular entity’ mentioned in the ClinicalTrials.gov.

The ‘investigational molecular entity’ of a clinical trial can be found in its title, description, and intervention in ClinicalTrials.gov. No standard controlled vocabulary was used, due to the lack of a standard terminology to describe novel investigational drugs. In addition, when investigating new indications for legacy drugs, it will give rise to heterogeneous naming for drugs and compounds if we do not use a standard vocabulary. A scenario of this sort is illustrated by clinical trials related to the drug Gleevec. A search of ClinicalTrials.gov with “Gleevec” carried out on dated May 17, 2020 retrieved 742 records. Many names such as Gleevec, Glivec, imatinib mesylate, and imatinib appeared in the search results. Although Gleevec and Glivec are the brand names and imatinib is the generic name of the drug, it appears that ClinicalTrials.gov users use all three terms interchangeably. The corresponding entities are however semantically distinct from the chemical informatics and drug manufacture’s perspectives. The Gleevec™ capsule is a drug product approved by FDA with a formulation composed of inactive ingredients and the active ingredient ‘imatinib mesylate’. (See Figure 4.) In addition, Gleevec has ‘imatinib’ as active moiety^4^. The ‘imatinib mesylate’ has ‘imatinib’ as a part, and ‘mesylate’ as a salt part. ‘Imatinib’, ‘imatinib mesylate’, ‘mesylate’ and ‘imatinib’ are all distinct compounds with assigned PubChem IDs. Figure 4 also shows an example of what it means to implement CTO to represent three clinical trials investigating the use of Gleevec, imatinib mesylate, and imatinib in treating patients with the three different conditions/diseases of COVID-19, scleroderma, and neurofibromatosis, respectively.

### 3.5. SCAIView Retrieval of COVID-19 Clinical Trial-Related Publications

Although, as stated by WHO^[3]^, making the content and results of clinical trials available to the public is a “scientific, ethical and moral responsibility”, the practice of publishing trial results is still marked by many deficiencies. The results of clinical trials as published in clinical trial registries may provide only high-level summaries. On the other hand, sponsors tend to publish trial protocols and/or results in depth in a scientific journal. In PubMed, one can retrieve primary (original) publications of clinical trials using the clinical trial registry identifiers. However, exhaustive search results cannot be achieved due to lack of annotations in reviews, in meta-analyses, or in older publications. SCAIView is an advanced search environment supporting semantic queries relating to biomedical entities that was developed at Fraunhofer SCAI, Germany. COVID-19 SCAIView (https://covid.scaiview.com) is currently under development to enable highly specific searches powered by CTO semantics on the COVID-19 Open Research Dataset (CORD-19)^[17]^, which is a subset of the SCAIView incorporated corpora. Published and maintained by Allen Institute for Artificial Intelligence, CORD-19 aggregates SARS-CoV-2, SARS and MERS related literatures from scattered sources, including PubMed, WHO, and pre-print platforms such as medRxiv and bioRxiv.

Currently, a text-mining engine implements a search algorithm to detect publications matching clinical trial registry identifier patterns stored in CTO. 2,039 documents were identified in the CORD-19 corpus. Of which, a total of 1,289 unique clinical study identifiers were found. Table 1 provides an overview of detected clinical trial identifiers for the specific clinical trial registries as on May 23, 2020.

Further analysis showed nearly 80% of the 2,039 publications referring to pre-COVID19 clinical trials of SARS, MERS, and ARDS. By filtering for the clinical trials starting in 2020, 469 publications referring to 177 COVID-19 related trials could be identified. The top 3 most cited clinical trials are NCT04252664 (cited in 23 publications), NCT04257656 (cited in 20), and ChiCTR2000029765 (cited in 17). Both NCT04252664 and NCT04257656 are Remdesivir RCT trials conducted in China and started in February 2020. ChiCTR2000029765 is an ongoing Chinese RCT trial investigating tocilizumab, a monoclonal antibody blocking IL-6, which is considered to play an important role in the Cytokine release syndrome (CRS) caused by SARS-CoV-2 that may lead to death.

Among all papers citing these three clinical trials, only NCT04257656 includes the published results of the trial; the others are mainly review papers, case reports, or commentaries. This may be due to the fact that the trials were suspended early, as in the case of NCT04252664, or to the fact that it is too early for publication of results. Overall, the utility of applying CTO in identify publications referring to COVID-19 clinical trials could be demonstrated. Further iterations to optimize the approach while continuously developing CTO are needed to improve performance.

## 4. Discussion

We presented our initial development of a community-based Clinical Trial Ontology (CTO) using an ontological realism approach. This initiative was driven by the need for greater interoperability between major clinical trial registries, and by two real-world use cases relating to PubChemRDF and SCAIView. CTO aims to provide a small set of ontologically engineered clinical trial specific terms for the ontology community. Many clinical trial-related standards, terminologies and vocabularies have been adopted in different circles and used in healthcare practice. These include CDISC, NCIt, and SNOMED CT.

Among the OBO Foundry ontologies, OBI, OPMI, OCRe, and ERO have defined many terms and relations relevant to clinical trial and clinical study research. Yet, no clinical trial focused and formally defined ontology has hitherto been available to the OBO Foundry community. Other previous non-BFO-compliant work include the PHUSE community’s Mini Study Ontology^[19,20]^, CTO-NDD^[10]^, and the Cochrane PICO Ontology (https://linkeddata.cochrane.org/pico-ontology). The PHUSE community focuses on representing submission data conformant to the CDISC standard in the RDF format, with no attention to semantics. None of these ontologies fits the needs of the use cases presented in this paper. In fact, the developers of OPMI and CTO-NDD themselves formed the CTO development team precisely to address their deficiencies. The CTO-NDD has therefore been refined to form part of the current CTO, and OPMI has donated multiple clinical trial-specific terms to CTO.

Although CTO has provided the basis for a simplified strategy for linking clinical trials to investigational drag and disease data in the PubChemRDF use case, and also provided synonyms and definitions for CTO-based text mining in SCAIView, challenges still remain for those who are developing this ontology. For example, *what is a clinical trial*? Traditionally, clinical trials are randomized, double-blind interventional studies in which both investigators and patients are unaware of which treatment is being administered. The ClinicalTrials.gov includes both interventional studies and observational studies because observational studies have been used for regulatory decision making. Furthermore, in the Framework for FDA’s Real-World Evidence Program (https://www.fda.gov/media/120060/download), FDA considers the traditional clinical trial as a type of clinical study. It is unclear if observational studies will in the future be considered as clinical trials given that they are non-interventional. In the CTO development team, how to ontologically represent and make distinctions between clinical study, clinical trial, and interventional and observational study is currently under active discussion.

The meanings of ‘study design’ and ‘study type’ are often ambiguous. CTO provides a distinct ontological treatment of study design as a plan specification (Figure 2). CTO contains many study design terms, such as allocation (e.g., randomized), intervention model (parallel assignment), primary purpose or masking (double blinded), cross-over design, factorial design, sequential, single arm, as well as study types including health services research, diagnostic test, basic science, prevention, prognosis study, screening, treatment study, epidemiological research, interventional clinical trial of medicinal product. These terms and relations among them have not as yet however been properly defined, and their hierarchies are under development in the current CTO.

Another challenge lies in the *BFO-realism* based approach, which has its roots in Aristotelian realism, and requires that the universals represented by the terms asserted in an ontology exist in space and time in their respective instances^[20]^. The definitions for terms in realism-based ontologies must adhere to very strict criteria. Each term must be provided with an Aristotelian definition which states the individually necessary and jointly sufficient conditions which must be satisfied be instances of the corresponding class^[21]^. These conditions are however not satisfiable in every case. Some difficult terms from this perspective might include the class of all those *things capable of being investigated in a clinical trial* (*e.g.* investigational drags, devices, vaccines, dietary supplement, behavioral treatment, and more) and the ‘*status*’ of a clinical trial. In clinical trials, besides ‘condition’ and ‘disease’, clinical trials might investigate also for instance ‘conditions’ or ‘quality of life’ or ‘health risk’. The OBO foundry has established that the universe ‘disease’ is a subclass of ‘disposition’; however, there is no adequate ontological agreement regarding use of terms such as ‘condition’, ‘quality of life’ and ‘health risk’. The status of a clinical trial, including ‘completed’, ‘recruiting’, ‘terminated’, ‘withdrawal’ and the like, is something that changes over time. By treating ‘status’ as a subclass of ‘realizable entity’ this issue can be addressed in a BFO conformant matter. However, a definition has still to be formulated that will reach a consensus among the CTO development team.

The further development of CTO will continue with its BFO-based approach and evolve as a community effort, thereby supporting WHO’s goal of further standardizing registration and reporting of clinical trials. Further applications of the ontology will also be explored, including application to study design, to results comparison across clinical trials, linking out to other data resources, and to the improvement in the development of specific text mining algorithms to identity relevant publications on specific clinical trials.

## Figures and Tables

**Table 1.** Number of clinical trials with references in publications from CORD-19 corpus

Clinical trial registry	Number of identified trials	Number of publications
ClinicalTrials.gov	1180	1823
Australian New Zealand Clinical Trials Registry	18	25
Chinese Clinical Trial Registry	53	142
German Clinical Trials Register	3	5
Iranian Registry of Clinical Trials	1	1
Japan Primary Registries Network	8	9
ISRCTN registry	18	23
EU Clinical Trials Register	1	3
Korean clinical trial registry (CRiS)	3	3
Pan African Clinical Trial Registry	1	2
Clinical Trials Registry - India	3	3

## Footnotes

4 Based on Code of Federal Regulations Title 21 (21 CFR) section 314.3, *active moiety* is the molecule or ion, excluding those appended portions of the molecule that cause the drug to be an ester, salt (including a salt with hydrogen or coordination bonds), or other noncovalent derivative (such as a complex, chelate, or clathrate) of the molecule, responsible for the physiological or pharmacological action of the drug substance.
